# Back pain reporting in young girls appears to be puberty-related

**DOI:** 10.1186/1471-2474-6-52

**Published:** 2005-11-01

**Authors:** Niels Wedderkopp, Lars Bo Andersen, Karsten Froberg, Charlotte Leboeuf-Yde

**Affiliations:** 1 The Back Research Center, BackCenter Funen, Lindevej 5, 5750 Ringe, Denmark; 2 Norwegian University of Sport and Physical Education, Sognsveien 220, 0806 Oslo, Norway; 3 Institute of Sportscience and Clinical Biomechanics, University of Southern Denmark, Campusvej 55, 5230 Odense M, Denmark

## Abstract

**Background:**

There is a large increase in back pain reporting in the early teens. In no previous study has the prevalence of low back pain been investigated in relation to the onset of puberty. The objective of this study was to establish whether the onset of puberty is associated with back pain reporting in young girls.

**Methods:**

A subsample of 254 girls aged 8–10 years and 165 girls aged 14–16 years from a cross-sectional survey of 481 children aged 8–10 years and 325 adolescents aged 14–16 years of both sexes.

Main outcome measures were back pain defined as low back pain, mid back pain, and/or neck pain in the past month.

Other variables of interest were Puberty (five different stages), age, body mass index, and smoking. Independent information on onset of puberty was obtained through a physical examination and on back pain through an individual structured interview. The association was studied between onset of puberty and the outcome variable (the one month period prevalence of back pain), controlling for overweight, and smoking. Odds ratios with 95% confidence intervals were used to describe bivariate associations, logistic regression with robust standard errors was used for multivariate analyses.

**Results:**

There is a highly significant trend for increased back pain reporting with increasing level of puberty until maturity is reached. The biggest leap appears between the second level (beginning of puberty) and the third level (mid puberty) and the findings remain after controlling for the covariates. These results emanate from the low back, whereas pain in the mid back and neck do not seem to be linked with pubertal stage.

**Conclusion:**

In girls, the reporting of low back pain increases in frequency during puberty until maturity, regardless of age. Why some girls are susceptible to back pain in the early stage of puberty is unknown.

## Background

Back pain has been shown to commence early in life [1,2]. In a previous report, it was shown that the one-month period prevalence of back pain (neck, mid back and/or low back pain) was 33% (95%CI: 29–37) for children aged 8–10 years and 47% (95% CI: 42–52) for adolescents aged 14–16 years [3]. Also the frequency of back pain has been shown to increase with age, particularly around the period of puberty [4].

Attempts have been made to identify the age, when back pain becomes common and, at least in the case of pain for the lower back, there seems to be a rapid increase in the back pain reporting around the age of puberty [5]. If the onset of back pain were a function of time, the consequence of the cumulative effects of injuries and repeated microtrauma, then it would be expected that back pain reporting commenced in boys, the gender most likely to incur injuries. However, it has previously been shown that that back pain reporting is more frequent in young girls than in young boys but that boys catch up within a year or two [5]. The reason for this should be sought in some sex (biological) or gender (behavioural) differences in that age group. Girls reach puberty earlier than boys. Therefore, to establish whether back pain reporting among the young is puberty-related, the prevalence of back pain was studied for girls attending school in grades 3 and 9 in relation to their stages of puberty and after controlling for smoking, and overweight.

## Method

The study design and methods have been extensively described elsewhere (**3**) and have been summarized in Appendix 1. A subsample of 254 female children and 165 female adolescents was selected from the participants in a randomly selected sample of Danish school-children attending elementary school (i.e. children aged 8–10 years and adolescents aged 14–16 years). These had been interviewed with help of a semi-structured questionnaire by one of the authors (NW) for the presence of back pain.

The one-month period prevalence of back pain was established by asking the pupils to point to any area of discomfort in the back (low back, mid back and/or neck region) reported to have occurred on the day of the study, in the week preceding the interview, or in the month preceding the interview, i.e. the one-month period prevalence of back pain.

Start of puberty was assessed according to Tanner [6] based on breast development. Pubertal development was graded in relation to breast development from stage 1 (not started puberty), through stage 2 (just starting), to stages 3–5, where 5 denotes that puberty has ended. Six of the girls refused to have their breasts examined resulting in a sample size of 413. The spread of data is shown in Table 1. Among the children, 29% of the young girls had started puberty vs. 99% of the adolescent girls.

**Table 1:** Stage of puberty by pain location according to sampling scheme, showing significant trend towards more low back pain and back pain anywhere with more advanced pubertal stage df = 1 for both calculations, chi-square = 42.9 and 17.29, p < 0.0001 for both calculations.

3^rd ^grade	Pain location	Puberty stage 1	Puberty stage 2	Puberty stage 3	Puberty stage 4	Puberty stage 5	Total (%)
	No pain	126	46	1	0	0	173 (68.7%)
	Low back pain	3	1	0	1	0	5 (2.0%)
	Mid back pain	30	15	1	1	0	47 (18.7%)
	Neck pain	19	5	2	0	0	26 (10.3%)
	Pain in two locations	0	1	0	0	0	1 (0.4%)
	Back pain anywhere	52	22	3	2	0	79 (31.4%)
	Total	178	68	4	2	0	252 (100%)

9^th ^grade							

	No pain	1	0	7	36	36	80 (49.7%)
	Low back pain	0	0	2	18	15	35 (21.7%)
	Mid back pain	0	0	3	12	14	29 (18.0%)
	Neck pain	0	0	0	6	4	10 (6.2%)
	Pain in two locations	0	0	0	2	5	7 (4.3%)
	Back pain anywhere	0	0	5	38	38	81 (50.3%)
	TOTAL	1	0	12	74	74	161

Data on back pain and puberty were collected by two independent examiners who were naïve in relation to the possible link between puberty and back pain, as the idea for the present project arose after the publication of a first set of articles, and the purpose of the main study was to determine various biological risk factors for cardiovascular disease.

One at the time, the four different definitions of back pain (back pain, low back pain, mid back pain, and neck pain) were cross-tabulated against the pubertal stage variable and reported as odds ratios with 95% confidence intervals.

These associations were tested for trends across ordered groups with a score test for trend of odds [7]. The score test for trend performs a test for linear trend of the log odds against the numerical code used for the exposure variable [7]. The association between overall back pain and puberty were, thereafter, studied using logistic regression, adjusting for overweight defined by BMI cut-points as specified by Cole et al.[8], and smoking, as these variables could be suspected to be associated to both back pain and puberty. Both the odds ratio of the individual puberty stages and the trend across the puberty stages were assessed.

To take into account the possible effect of cluster sampling (independence of observations across groups but not necessarily independence within groups), "robust standard errors" were used in the logistic regression. These have the ability to relax the assumption of independence of the observations, i.e. they can produce "correct" standard errors (in the measurement sense) even if the observations are correlated [9]. We chose to do multivariate analysis only on the back pain anywhere group because this was the only group with enough subjects for this type of analysis (Table 1).

To test the goodness of fit, the Hosmer Lemeshaw goodness of fit statistics was applied to logistic regression model [10].

## Results

The spread of data by pubertal stage is shown in Table 1. As can be seen in Table 2 there is a bivariate trend for increase for reporting of back pain anywhere (p < 0.0001) and low back pain (p < 0.0001) with increasing level of puberty until maturity is reached. The same trend is found after having adjusted for smoking and overweight. Both in the bivariate analysis and after having controlled for possible confounders, the biggest leap appears between the second level (beginning of puberty) and the third level (mid puberty). The association was tested for back pain anywhere controlling for the potential confounders (overweight and smoking), and it was noted that the above results held (Table 3.). Test for goodness of fit showed good fit of the model, with p = 0.97. In addition logistic regression was used to test for trend when adjusting for the possible confounders, resulting in a significant odds ratio of 1.2 (95% CI 1.2 ; 1.4).

**Table 2:** The bivariate associations between pubertal stage and a) back pain anywhere b) low back pain, c) mid back pain, and d) neck pain reported as odds ratios and 95% confidence intervals.

Definition of back pain	Puberty stage 1 (index group)	Puberty stage 2	Puberty stage 3	Puberty stage 4	Puberty stage 5
Back pain anywhere	1	1.1 0.6–2.0	2.3 0.8–6.5	2.6 1.5–4.7	2.4 1.4–4.3
Low back pain	1	0.9 0.1–8.5	8.2 1.2–55.6	19.6 5.0–76.3	14.7 3.8–56.6
Mid back pain	1	1.4 0.7–2.8	1.6 0.5–5.4	1.0 0.5–2.1	1.1 0.6–2.3
Neck pain	1	0.7 0.2–1.8	1.2 0.2–5.6	0.7 0.3–1.9	0.5 0.2–1.4

**Table 3:** The multi variable association between pubertal stage and back pain anywhere, adjusting for overweight and smoking using logistic regression, reported as odds ratios with 95% confidence intervals.

Definition of back pain	Puberty stage 1 (index group)	Puberty stage 2	Puberty stage 3	Puberty stage 4	Puberty stage 5
Back pain anywhere	1	1.1 0.7–2.0	1.6 0.5–4.6	2.0 1.3–3.5	2.1 1.1–4.1

The associations between back pain anywhere and the covariates expressed as odds ratios were: 2.9 (95% CI 1.3 ; 6.5) for smoking and 0.7 (95% CI 0.3 ; 1.7) for overweight.

## Discussion

To our knowledge, this is the first time that the presence of back pain is studied in relation to pubertal stage. The results indicate that back problems and back pain might be related to puberty stage.

Two possibilities related to puberty spring to mind. First, the growth spurt initiated during puberty could be the initiating factor of back pain and back problems, as has been suggested by others [11,12]. For example Salminen et al. found a relationship between rapid growth, degenerative changes in the spine, and back pain in adolescents [11] and, in a Canadian study a significant association could be found between high growth spurt and the development of adolescent musculoskeletal pain over a 1-yr period [13]. This could also explain why the increase in back pain in our population occurs at puberty stages 3 and 4, as it is at these stages that the growth spurt occurs, which might make the back more susceptible to mechanical injuries. On the other hand, over a period of one year, increase in height in 4^th ^grade Finnish children was not found to be associated with self-reported low back pain [14]. Obviously it should be further investigated what circumstances in the early stages of puberty can induce increased back pain reporting in girls.

We found one study, in which low back- and neck/shoulder pain was studied in relation to whether the menarche started early, average or late in 14, 16 and 18 year old Finnish adolescents [14]. A weak association was found with early onset of puberty after having controlled for age and sex. Unfortunately the study design did not allow for an assessment of pain in relation to the different stages of puberty.

Second, hormone-induced changes at puberty might have an effect on the attitudes to or perception of pain. We could find no information as to any evidence for or against such suppositions. Nonetheless, the perception of experimentally induced pain was noted to fluctuate across the menstrual cycle in females [15] and pain perception in women has been shown to be different to that in men [16].

We decided not to include physical activity, age and height in the analysis although we have access to that information in our dataset. The reasons were that we have shown earlier that in this cohort objectively measured physical activity was not related to back pain [17], and preliminary analyses revealed that both age and height were closely related to puberty level and created collinearity problems.

The results should and have been interpreted with caution for two reasons. First, it is a cross sectional study and a secondary analysis. Our title: "Back pain reporting in young girls appears to be puberty-related", illustrates that the results derive from a hypothesis generating analysis rather than confirmatory analysis. To be able to perform a confirmatory analysis, a prospective study, including both genders from age 8 to 18 with frequent follow-ups, would be required. Second, when stratifying on the different spinal regions and puberty the number of subjects in the calculations gets small, and the odds ratios could therefore be inflated. But as the trend in the results is the same, in the analyses of back pain anywhere alone and of low back pain across the puberty stages, we believe that the association is true.

Ideally, we should have made the same analyses on the boys, as data on these were collected. However, we found a systematic error, as the distribution of boys having started puberty was illogical, 85% being classified as having reached end of puberty, in comparison with only 43% of the girls. As girls generally start and end puberty before boys and the end of puberty in boys usually is around age 18, the data on puberty in boys could not be included in the study. It therefore now remains to find out if back pain reporting in boys is also linked with puberty and whether back pain is simply an inevitable aspect of growing up.

## Appendix 1. Description of study design and method

### Participants

#### Target population

8–10-yr old children and 14–16-yr old adolescents, attending state schools in Odense, Denmark, and their parents were invited to participate in a cardiovascular study.

#### Sampling

Schools were stratified according to type (age ranking, selection procedures, single/mixed sex), location (urban, suburban, rural), and socio-economic uptake of area. Proportional, two-stage cluster sample of children from each stratum. Primary units (clusters): schools; secondary units: children in 3^rd ^and 9^th ^grades. Children were randomly selected using random number tables.

#### Study sample

Agreed to participate: 25/28 schools (89%) and 1020/1356 child-parent pairs (75%). Of these, 806 were included in the present study.

#### Representativeness

The distribution of parental income and educational levels were representative of the national distribution (Source for comparison: Information from the Danish Central Statistical Office, Copenhagen).

### Data collection

#### Design and ethics

Questionnaire data were collected from parents, interviews conducted with children, and physical measurements made of children in a cross-sectional survey. The study was approved by the local ethics committee.

#### Questionnaire

Questionnaires were handled through teachers and by mail for non-responders. They contained: information on parental health, cardiovascular disease, cardiovascular risk factors, and socioeconomic status, plus information on children's birth weight and chronic diseases.

#### Interview

Back pain interviews were conducted by NW and tested for face validity. Children were asked if they had any back or neck pain within the preceding month and whether the back pain had any consequences.

#### Physical examination

Height, weight, body fatness and stage of puberty were measured but not all used in the present report.

#### Validity

None of the variables was validated against a golden measure.

### Manipulation of data

#### Data entry and quality control

Data entry was manual, checked by a second person. There was an additional verification and, if necessary, correction of outliers but none was removed from the data set.

## Competing interests

The author(s) declare that they have no competing interests.

## Authors' contributions

NW and CLY developed the aim and analytical approach for this paper. NW, LBA and KF were responsible for elements of the study design specific to this analysis. KF, NW and LBA obtained funding, co-ordinated and performed data collection. NW undertook the statistical analyses and NW and CLY wrote the first draft of the paper. All authors contributed to the final version.

NW and CLY act as guarantors.

## Pre-publication history

The pre-publication history for this paper can be accessed here:


